# Cytotoxicity towards human endothelial cells, induced by neutrophil myeloperoxidase: protection by ceftazidime

**DOI:** 10.1155/S0962935195000706

**Published:** 1995-11

**Authors:** M. Mathy-Hartert, G. Deby-Dupont, C. Deby, L. Jadoul, A. Vandenberghe, M. Lamy

**Affiliations:** 1 Centre for the Biochemistry of Oxygen Institut de Chimie, B6 Université de Liège Domaine Universitaire du Sart Tilman Liège 4000 Belgium; 2 Department of Anesthesiology and Intensive Care Medicine Centre Hospitalier Universitaire, B35 Domaine Universitaire du Sart Tilman Liège 4000 Belgium; 3 Glaxo Belgium sa Boulevard du Triomphe, 172 Bruxelles Belgium

## Abstract

We investigated the effects of the antibiotic ceftazidime (CAZ) on the cytolytic action of the neutrophil myeloperoxidase–hydrogen peroxide–chloride anion system (MPO/H_2_O_2_/Cl^−^). In this system, myeloperoxidase catalyses the conversion of H_2_O_2_ and CI^−^ to the cytotoxic agent HOCl. Stimulated neutrophils can release MPO into the extracellular environment and then may cause tissue injury through direct endothelial cells lysis. We showed that human umbilical vein endothelial cells (HUVEC) were capable of taking up active MPO. In presence of H_2_O_2_ (10^−4^ M), this uptake was accompanied by cell lysis. The cytolysis was estimated by the release of ^51^Cr from HUVEC and expressed as an index of cytotoxicity (*IC*). Dose dependent protection was obtained for CAZ concentrations ranging from 10^−5^ to 10^−3^ M;this can be attributed to inactivation of HOCl by the drug. This protection is comparable to that obtained with methionine and histidine, both of which are known to neutralize HOCl. This protection by CAZ could also be attributed to inactivation of H_2_O_2_, but when cytolysis was achieved with H_2_O_2_ or O_2_^-^ generating enzymatic systems, no protection by CAZ was observed. Moreover, the peroxidation activity of MPO (action on H_2_O_2_) was not affected by CAZ, while CAZ prevented the chlorination activity of MPO (chlorination of monochlorodimedon). So, we concluded that CAZ acts via HOCl inactivation. These antioxidant properties of CAZ may be clinically useful in pathological situations where excessive activation of neutrophils occurs, such as in sepsis.


Mediators of Inflammation 4, 437–443 (1995)

### Cytotoxicity towards human endothelial cells, induced by neutrophil myeloperoxidase: protection by ceftazidime

M. Mathy-Hartert,[1,CA] G. Deby-Dupont,[1,2] C. Deby,[1] L. Jadoul,[3] A. Vandenberghe[3] and M. Lamy[1,2]

[1]Centre for the Biochemistry of Oxygen, Institut de Chimie, B6, Université de Liège, Domaine Universitaire du Sart Tilman, 4000 Liège; [2]Department of Anesthesiology and Intensive Care Medicine, Centre Hospitalier Universitaire, B35, Domaine Universitaire du Sart Tilman, 4000 Liège; and [3]Glaxo Belgium sa, Boulevard du Triomphe, 172, Bruxelles, Belgique

[CA]Corresponding Author

**We investigated the effects of the antibiotic ceftazidime (CAZ) on the cytolytic action of the neutrophil myeloperoxidase–hydrogen peroxide–chloride anion system (MPO/$H_2O_2$/$Cl^-$). In this system, myeloperoxidase catalyses the conversion of $H_2O_2$ and $Cl^-$ to the cytotoxic agent HOCl. Stimulated neutrophils can release MPO into the extracellular environment and then may cause tissue injury through direct endothelial cells lysis. We showed that human umbilical vein endothelial cells (HUVEC) were capable of taking up active MPO. In presence of $H_2O_2$ ($10^{-4}$ M), this uptake was accompanied by cell lysis. The cytolysis was estimated by the release of $^{51}Cr$ from HUVEC and expressed as an index of cytotoxicity (*IC*). Dose dependent protection was obtained for CAZ concentrations ranging from $10^{-5}$ to $10^{-3}$ M; this can be attributed to inactivation of HOCl by the drug. This protection is comparable to that obtained with methionine and histidine, both of which are known to neutralize HOCl. This protection by CAZ could also be attributed to inactivation of $H_2O_2$, but when cytolysis was achieved with $H_2O_2$ or $O_2^-$ generating enzymatic systems, no protection by CAZ was observed. Moreover, the peroxidation activity of MPO (action on $H_2O_2$) was not affected by CAZ, while CAZ prevented the chlorination activity of MPO (chlorination of monochlorodimedon). So, we concluded that CAZ acts via HOCl inactivation. These antioxidant properties of CAZ may be clinically useful in pathological situations where excessive activation of neutrophils occurs, such as in sepsis.**

**Key words:** Antibiotics, ceftazidime, endothelial cells, hypochlorous acid, myeloperoxidase, oxidant stress

### Introduction

Sepsis, septic shock, severe trauma, hypovolaemic shock and acute pancreatitis are examples of severe pathological situations accompanied by an intense inflammatory reaction involving activation of specific cells and release of mediators. This inflammatory reaction occasionally overwhelms the organism's defences, with excessive activation of polymorphonuclear leucocytes (PMN). This can lead to the systemic inflammatory response syndrome (SIRS) and eventually to multiple organ dysfunction syndrome (MODS).[1–3] PMN activation produces activated oxygen species (mainly superoxide anion and hydrogen peroxide, $H_2O_2$), inflammatory mediators, and proteolytic and hydrolytic enzymes.[4,5] PMN also release myeloperoxidase (MPO) from their granules; in the presence of $H_2O_2$ and chloride anion ($Cl^-$), MPO generates hypochlorous acid (HOCl). HOCl reacts with amines to form chloramines, but also with $H_2O_2$ itself to produce singlet oxygen, an activated form of oxygen.[6,7] These activated oxygen species and oxidant products of MPO activity normally play a beneficial role in host defence against invading microorganisms. However, their excessive production can be detrimental for tissues, especially for endothelial cells, such as, when PMN are trapped in capillaries and become strongly adherent to endothelium.[8] They are also destructive for plasma proteins, especially those with thiol (–SH) functions, such as $\alpha_2$ macroglobulin, an essential plasmatic antiproteinase.[9–11] In these situations associated with excessive activation of PMN, therapeutic agents capable of neutralizing these active oxygen species and protecting endothelial cells from oxidant stress would have potential clinical utility. Recent research has demonstrated that antibiotic molecules of the aminoglycoside and

© 1995 Rapid Science Publishers cephalosporin families can protect epithelial cells submitted to an oxidant stress, and are scavengers for HOCl molecules.[12–14]

We have demonstrated that the antibiotic ceftazidime (CAZ), which belongs to the cephalosporin family, exerts an antioxidant effect *in vitro*: it inhibits perferryl mediated lipoperoxidation by iron scavenging, neutralizes HOCl, and quenches singlet oxygen.[15–17] The purpose of this study was to demonstrate that CAZ is able to protect endothelial cells from the oxidant stress induced by the activity of MPO or by HOCl. In our study, CAZ was used as a model of broad spectrum β-lactamase resistant antibiotics, which are widely used in clinical situations of severe sepsis, as mentioned above; these are precisely the situations wherein intense PMN activation is seen.

### Materials and Methods

*Cell culture:* Human umbilical vein endothelial cells (HUVEC) were isolated by collagenase treatment of umbilical cords according to Jaffe *et al.*[18] HUVEC were cultured on 0.2% gelatine coated dishes in M199 medium supplemented with 10% heat-inactivated foetal calf serum (Gibco), 5% heat-inactivated human serum (Sigma), penicillin (100 U/ml), streptomycin (100 μg/ml), heparin (90 μg/ml) and endothelial cell growth factor (Boehringer–Manheim) (20 μg/ml). Adherent HUVEC in six multiwell plates (passage 2) were used in all experiments.

*Assay for MPO enzyme activity:* MPO was purified from human polymorphonuclear neutrophils as described previously.[19,20] Its enzyme activity was determined by spectrophotometric methods.

MPO peroxidative activity. MPO peroxidative activity was assayed by measuring the absorbance increase at 460 nm caused by the oxidation of ortho-dianisidine.[21] MPO (dissolved in 50 μl) was added to 3 ml of 50 mM ortho-dianisidine in Sörensen buffer pH 5.5 and the reaction was started by the addition of $H_2O_2$ at a final concentration of 0.15 mM. The absorbance increase at 460 nm was followed for 1 min. One unit of activity was defined as the amount of MPO which produced an absorbance increase of 1 optical density unit per minute.

MPO-dependent chlorination activity. MPO-dependent chlorination activity was measured by following the conversion of monochlorodimedon to dichlorodimedon at 290 nm.[22] Typical experimental conditions were as follows: MPO (5 μg) was incubated in 3 ml of monochlorodimedon (24 μM) in 100 mM phosphate buffer (pH 5.5) with NaCl (50 mM) added. The reaction was started by the addition of $H_2O_2$ (0.1 mM) and the decrease of absorbance at 290 nm was followed for 1 min. One unit of activity was defined as the amount of MPO which produced an absorbance decrease of 1 optical density unit per minute.

To determine the effects of CAZ on MPO enzyme activities, MPO was incubated with different concentrations of CAZ for 5 min at room temperature before the enzyme assays. The data represent arithmetic means ± S.D. of triplicates.

*MPO uptake by HUVEC:* Confluent cells in 1 ml of Hanks' Balanced Salt Solution (HBSS) were incubated at 37°C with increasing amounts of MPO (from 0 to 30 μg) for 3 h or with a fixed amount of MPO (5 μg) for periods of time ranging from 0 to 360 min. Each assay was done in triplicate and the entire experiment was repeated three times. After these incubations, cells were washed three times with HBSS, and the MPO content of the cells was estimated by measurement of MPO peroxidative activity (see above). Ortho-dianisidine (1 ml) was added to adherent cells and the reaction was started by the addition of $H_2O_2$ at a final concentration of 0.15 mM. After an incubation of 30 min, the reaction buffer was collected and the absorbance at 460 nm was read. The amount of MPO present in the cells was calculated from a standard curve for which known amounts of MPO were incubated under the same conditions.

*Cytotoxicity assay:* Cytotoxicity was assessed by measuring the release of previously incorporated $^{51}Cr$.[23] Confluent HUVEC in six multiwell plates were labelled overnight by the addition of 20 μCi $^{51}Cr$ in culture medium per well (sodium chromate, Amersham). HUVEC were washed in HBSS to remove unincorporated $^{51}Cr$ and the cells were then submitted to oxidant stress as described below. Each assay was done in triplicate. At the end of the oxidant stress period, the supernatants were collected and the cells were washed three times with HBSS. Supernatant and washings were pooled and $^{51}Cr$ release [*R*] was quantified by γ counting.

Cells were lysed in NaOH (1 N) and the intracellular $^{51}Cr$ [*I*] was counted. The percentage of $^{51}Cr$ release [*PR*] was calculated for each test condition as follows:

$$PR = \frac{R}{R + I} \times 100$$

Where $R + I$ is the total amount of $^{51}Cr$ present in the cells before the application of the oxidant stress. An index of cytotoxicity [*IC*] was calculated according to the following formula:

$$IC = 100 \times \frac{[PR]_{test} - [PR]_{cont}}{100 - [PR]_{cont}}$$

where $[PR]_{cont}$ represents the spontaneous release of $^{51}Cr$ (in percent as above) by the control cells incubated in HBSS alone.

*Oxidant stress against HUVEC:* Four oxidant stressors were used against HUVEC. For each oxidant stress assay, controls were performed in which HUVEC were incubated under the same conditions in HBSS alone; each assay was done in triplicate.

*Cytotoxicity induced by the MPO/$H_2O_2/Cl^-$ system.* HUVEC in 1 ml of HBSS were incubated in the presence of MPO (5 μg) at 37°C for 2 h. The $Cl^-$ required for MPO enzyme activity was supplied by HBSS. $H_2O_2$ ($10^{-4}$ M) was added to initiate MPO enzyme activity and after a further 2 h incubation at 37°C, *IC* was determined as described before.

*Cytotoxicity induced by sodium hypochlorite (NaOCl).* NaOCl ($10^{-3}$ M) was added to HUVEC in 1 ml of HBSS. After a 2 h incubation, *IC* was evaluated.

*Cytotoxicity induced by the glucose/glucose oxidase system.* HUVEC in 1 ml HBSS (containing 5 mM of glucose) were incubated overnight with glucose oxidase (0.2 U) (Boehringer–Manheim) prior to *IC* evaluation.

*Cytotoxicity induced by the xanthine/xanthine oxidase system.* HUVEC in 1 ml of HBSS were treated with 2 mM xanthine/20 mU of xanthine oxidase (Boehringer–Manheim) for 2 h following which *IC* was determined.

*Protection from oxidant stress:* The effects of CAZ on oxidant stress were compared to methionine, histidine and dabco (1,4-diazabicyclo[2.2.2]octane), which are well known HOCl and $^1O_2$ scavengers.[24,25] $^{51}Cr$ labelled HUVEC in 1 ml of HBSS supplemented with one of the putative protectors (CAZ, methionine, histidine or dabco) were incubated for 2 h prior to initiating the cytotoxic treatments described above. For the oxidant stress with MPO/$H_2O_2/Cl^-$, the protector was added with MPO 2 h before the addition of $H_2O_2$. After the stress, *IC* was determined, and the % protection was obtained according to the following formula:

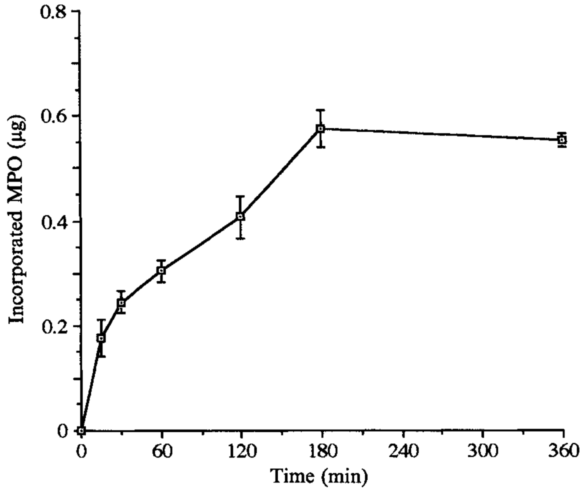

FIG. 1. Kinetics of MPO uptake by HUVEC (4 × $10^5$ cells). Confluent HUVEC were incubated with 5 μg of MPO. After cell washings, MPO uptake was estimated by the ortho-dianisidine assay (see text). Data represent arithmetic mean ± S.D. of triplicates.

$$\%\ \text{protection} = 100 \times \left(1 - \frac{IC_{eff}}{IC_{stress}}\right)$$

where $IC_{eff}$ and $IC_{stress}$ were, respectively, the *IC* obtained in the presence or in the absence of the study drug.

### Results

*MPO uptake by HUVEC:* The results for the kinetics of MPO uptake are presented in Fig. 1. MPO was quickly taken up by HUVEC; after only 15 min of incubation 0.177 ± 0.037 μg of MPO were already present in the cells. The maximal uptake reached a plateau after 180 min, with 0.553 ± 0.012 μg taken up. This maximal value corresponds to an uptake of 11.00 ± 0.24% of added MPO.

The dose–response curve of MPO uptake by HUVEC is shown in Fig. 2. The uptake of MPO reached a plateau for a MPO dose of 7.5 μg of MPO. At this dose, 0.585 ± 0.018 μg of MPO was taken up by the HUVEC; this corresponded to an uptake of 7.80 ± 0.24% of added MPO.

*Cytotoxicity of the MPO/$H_2O_2/Cl^-$ system on HUVEC:* When HUVEC were incubated for 2 h with MPO (5 μg) and $H_2O_2$ ($10^{-4}$M) added simultaneously, only a low cytotoxicity ($IC = 5.04 \pm 2.1$) was found. No cytotoxicity was obtained with either MPO or $H_2O_2$ alone. To permit the uptake of MPO prior to initiating the oxidant stress with $H_2O_2$, the cells were pre-incubated with MPO for 2 h. This time of pre-

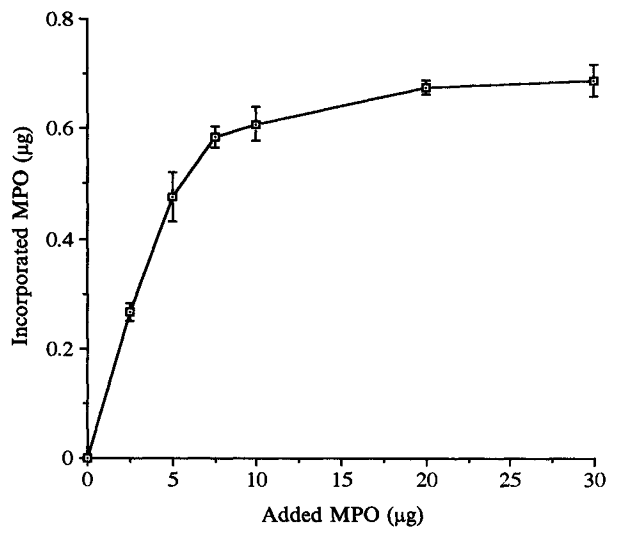

FIG. 2. Dose-response curve of MPO uptake by HUVEC ($4 \times 10^5$ cells). Adherent HUVEC were incubated for 3 h at 37°C with increasing amounts of MPO (0 to 30 µg). After cell washings, MPO uptake was estimated by the ortho-dianisidine assay (see text). Data represent arithmetic mean ± S.D. of triplicates.

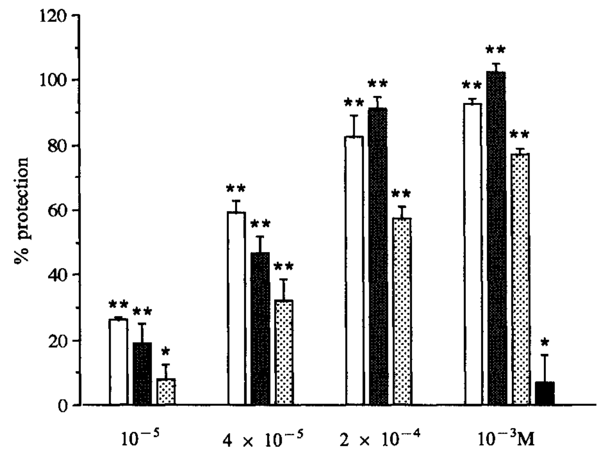

FIG. 4. Protection of HUVEC from $MPO/H_2O_2/Cl^-$ stress. Comparison of ceftazidime (CAZ) with methionine, histidine and dabco. $^{51}Cr$ labelled HUVEC were preincubated with MPO (5 µg) and variable concentrations of the protectors for 2 h at 37°C: □, CAZ; ▩, methionine; ▒, histidine; ■, dabco. $H_2O_2$ ($10^{-4}$ M) was added to initiate stress and the % protection was calculated after 2 h. The results are expressed as arithmetic mean ± S.D. of three experiments performed in triplicate. *No statistical difference ($p > 0.05$) and **statistical difference ($p < 0.05$) *vs.* stress without protector when analysed with the two-tailed Student's *t*-test.

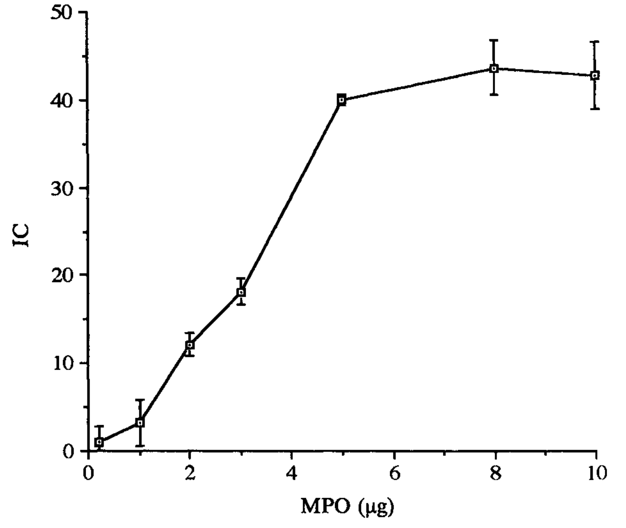

FIG. 3. Dose-response of MPO cytotoxicity on HUVEC. Confluent HUVEC were preincubated with increasing doses of MPO for 2 h. Oxidant stress was then induced by addition of $H_2O_2$ and the cytotoxicity index (*IC*) was determined after 2 h incubation (see text). Data represent mean ± S.D. of triplicates.

incubation was chosen because after 2 h MPO uptake was near its maximal value, but still on the linear part of the curve of Fig. 1. After addition of $H_2O_2$ ($10^{-4}$M), a further incubation of 2 h with the complete cytotoxic system ($MPO/H_2O_2/Cl^-$) was performed. After this time of incubation, convenient and reproducible *IC* were obtained. For shorter times of incubation, we measured low *IC* values, insufficient to allow reproducible assays of putative protective substances.

Figure 3 plots the dose-response effect of MPO on HUVEC cytotoxicity: a plateau was reached at 5 µg MPO (with a maximal $IC = 39.92 \pm 0.64$).

*Protection of HUVEC from $MPO/H_2O_2/Cl^-$ or NaOCl oxidant stresses:* To test the effects of putative protectors (CAZ, methionine, histidine, or dabco), the protector was added at different concentrations on HUVEC, together with 5 µg of MPO, and the cells were incubated for 2 h prior to addition of $H_2O_2$ (Fig. 4). We controlled the effects of CAZ on MPO uptake by endothelial cells and found neither inhibiting nor enhancing effects of the antibiotic: without CAZ, we found an uptake of 0.476 ± 0.044 µg MPO, and with CAZ ($10^{-3}$M), an uptake of 0.469 ± 0.011 µg (no statistical difference). After the stress, *IC* was calculated and the results were expressed as percent protection according to the formula defined in Materials and Methods: a protection of 100% corresponds to a total inhibition of stress-induced cytotoxicity.

The protection by CAZ was dose-dependent and particularly effective at the concentration of $10^{-3}$M (percent protection = 92.4 ± 1.7). This protection was confirmed by light microscope observations, which indicated a similar cellular aspect for control cells (without stress) and cells

**Table 1.** CAZ protection of HUVEC from NaOCl stress. Comparison with methionine and histidine

| Protector | % protection |
|---|---|
| CAZ ($10^{-3}$ M) | 77.90 ± 11.50 |
| Methionine ($10^{-3}$ M) | 91.38 ± 5.20 |
| Histidine ($10^{-3}$ M) | 20.18 ± 12.00 |

The data represent mean ± S.D. of three experiments performed in triplicate stressed in the presence of CAZ $10^{-3}$ M, while the cells stressed in the absence of CAZ appeared strongly damaged (loss of most of the cells and strong morphologic changes). At a CAZ concentration of $10^{-5}$ M, a protective effect was still observed (percent protection = 26.0 ± 0.9). Methionine and CAZ demonstrated comparable protective effects (no statistically significant difference between the two compounds), but histidine was less effective ($p < 0.05$ for each concentration compared to CAZ and methionine). In contrast, no protection was obtained with dabco (percent protection = 6.6 ± 9.0).

From these results, we concluded that CAZ was capable of neutralizing cytotoxicity induced by the MPO/$H_2O_2$/$Cl^-$ system by inactivating the HOCl generated by MPO. To confirm the direct effect of CAZ on NaOCl, $^{51}Cr$ labelled HUVEC were exposed to NaOCl stress in presence of CAZ, methionine, or histidine; the cells were incubated at 37°C for 2 h in the presence of putative protector (at a concentration of $10^{-3}$ M). NaOCl ($10^{-3}$ M) was then added and the *IC* was determined after a further 2 h incubation. The *IC* in the absence of protector was 14.86 ± 3.15 and the percent protection, calculated as previously described, are presented in Table 1. Marked protection was seen with CAZ and methionine, while histidine was weakly active.

*Absence of a protective effect of CAZ on oxidant stress generating $H_2O_2$:* To test the effect of CAZ on HUVEC cytotoxicity induced by $H_2O_2$ or $O_2^-$, we incubated the cells in the presence of enzymatic systems generating this reactive oxygen species: glucose/glucose oxidase or xanthine/xanthine oxidase systems.

For the two stresses, no statistically significant difference was observed (two-tailed Student's test) between the cytotoxicity obtained in the absence or in the presence of CAZ ($10^{-3}$ M). The *IC* values obtained for glucose/glucose oxidase stress were respectively 25.02 ± 3.91 without CAZ and 25.93 ± 5.26 with CAZ. For xanthine/xanthine oxidase stress the *IC* values were 76.98 ± 0.49 without CAZ and 78.37 ± 2.31 with CAZ.

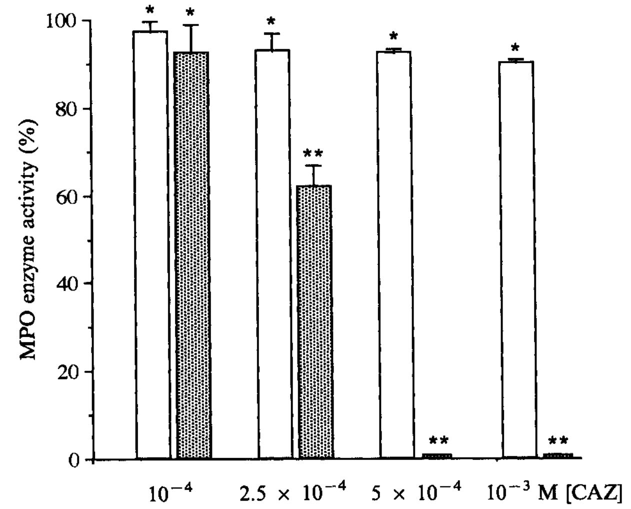

FIG. 5. Effects of CAZ on MPO enzyme activities. MPO was preincubated with different concentrations of CAZ for 5 min at room temperature. □, peroxidative activity of MPO; ▩, chlorination activity of MPO. MPO enzyme activities obtained in the absence of CAZ were taken as the reference (100%). Each point represents the mean ± S.D. of two experiments performed in triplicate. *No statistical difference ($p > 0.05$) and **statistical difference ($p < 0.05$) *vs.* control when analysed with the two-tailed Student's *t*-test.

*Effects of CAZ on MPO enzyme activities:* Even at concentrations of CAZ ($10^{-3}$ M) which were highly protective for HUVEC submitted to MPO/$H_2O_2$/$Cl^-$ stress, only weak inhibition of MPO peroxidative activity was observed (90.0 ± 0.7% of control activity) (Fig. 5). In contrast, MPO-dependent chlorination of monochlorimedon was very sensitive to CAZ: no chlorination was observed in the presence of $5 \times 10^{-4}$ M and only 61.0 ± 4.9% of activity was conserved in the presence of $2.5 \times 10^{-4}$ M CAZ.

### Discussion

In this study we demonstrate that endothelial cells are capable of binding and taking up myeloperoxidase. This uptake shows steep time dependency at 37°C, and is maximal at 3 h incubation. The yield of the uptake process is low, with a maximum incorporation of 11% when 5 μg of MPO are added to the culture medium (Fig. 1). Increasing the amount of MPO added to the medium does not increase uptake, which peaks at 7.5 μg (62.4 nM) added MPO. At this level, 7.8% of the added dose is incorporated after 3 h incubation. These results agree with those of Zabucchi *et al.*[26] who show that 75% of endothelial cells have a positive cytochemical peroxidase reaction after preincubation with high concentrations of MPO (up to 420 nM). These workers also showed that the enzyme was present on the cell membrane and in the cytoplasm. Using the same cytochemical reaction, we showed uptake of MPO by endothelial cells, and its presence in intracellular granules (results not shown). Spectrophotometric measurement of intracellular enzymatic activity confirmed uptake and showed that the internalized enzyme retained its activity. We used lower concentrations of MPO than Zabucchi *et al.*, but in our study, we used a 3 h incubation (as opposed to the 10 min used by Zabucchi's group). This duration of incubation did not alter the enzyme, because at the end of the period, the sum of enzyme left in the supernatant and that incorporated into the cells exactly equalled the total initially added.

In the presence of $H_2O_2$, MPO produces a cytolytic oxidative stress on endothelial cells, which we have expressed as a cytotoxicity index. This *IC* increases linearly with the dose of MPO with a plateau at 5 μg per 4 × $10^5$ cells. No cytotoxicity is observed, however, if MPO is not pre-incubated with the cells prior to addition of $H_2O_2$. This indicates that incorporation of the enzyme (or its absorption to the cell surface) is necessary. In subsequent experiments of the protective effects of added substances, we used a concentration of 5 μg for 4 × $10^5$ cells. We chose a 2 h pre-incubation at 37°C, because this timing allowed reproducible uptake across batches of cells. After addition of $H_2O_2$, a further 2 h incubation was chosen because this timing allowed a convenient and reproducible cytotoxicity.

Several antibiotics have been reported to have antioxidant properties against active forms of oxygen.[14,27–29] We chose to use ceftazidime because it is a highly effective agent, a broad-spectrum β-lactamase resistant antibiotic with anti-*Pseudomonas* activity, widely used in patients in the intensive care unit. CAZ protects endothelial cells from the oxidant stress induced by MPO in a dose dependent fashion, comparably to methionine, and more powerfully than histidine. These two amino acids are capable of scavenging HOCl, formed by the enzyme action of MPO in the presence of $H_2O_2$ and $Cl^-$.[4,5] The protective effect of CAZ could thus be attributed to its reaction with HOCl, neutralizing the oxidant activity responsible for the cytolysis seen in our experimental model. This mode of action is confirmed by the observation of a protective effect of CAZ on endothelial cells against direct attack by added NaOCl. This protection is less than that of methionine, but superior to that of histidine. This latter compound is less active on NaOCl stress than on $MPO/H_2O_2/Cl^-$ stress. This can be explained by the differences in the two types of stress. In the first case, the concentration of HOCl directly reaches $10^{-3}$ M. The activity of MPO induces a progressive release of low concentrations of HOCl, without reaching a final concentration of $10^{-3}$ M. The reaction of CAZ with HOCl occurs simultaneously with its formation by MPO, and prevents its chlorination of monochlorodimedon. On the other hand, CAZ has no effect on the peroxidase activity of MPO. The reaction of CAZ with HOCl could occur at either thioether groups or near amine groups of the antibiotic. It should be noted, however, that according to Lapenna *et al.*,[14] the reaction of CAZ with HOCl does not lead to formation of chloramines, which pleads against involvement of amine groups. The protective effect of methionine confirms a role of sulfur atoms, while histidine's action can only be explained by the presence of nitrogen, with the formation of non-toxic chloramines. The lack of a sulfur atom in histidine could partially explain the lower protective effect of this compound compared to CAZ and methionine.

In our model, neutralization of HOCl by CAZ is the principal mode of action of the antibiotic. The simultaneous presence of $H_2O_2$ and HOCl during the activity of MPO could lead to the formation of singlet oxygen ($^1O_2$), a highly reactive species.[30–32] We have previously shown that CAZ is capable of interacting with $^1O_2$, deactivating it more powerfully than dabco, a well-characterized quencher.[15–17,25] Under our experimental conditions, dabco at a concentration of $10^{-3}$ M was only weakly protective. This would seem to indicate that singlet oxygen is not the principal source of oxidant stress on endothelial cells induced by the activity of MPO. On the other hand, the lack of efficacy of CAZ against oxidant stress induced by glucose oxidase, and especially xanthine oxidase, which both produce superoxide anion and $H_2O_2$, indicates that the antibiotic does not act directly on these two activated species of oxygen, what has been already demonstrated for other antibiotics.[33]

Cantin *et al.*[13] described similar cytotoxicity of the $MPO/H_2O_2/Cl^-$ system against epithelial cells, using 2.5 μg/ml MPO, and observed a protective effect of CAZ at concentrations approximately equal to those we used. Ottonello *et al.*[12] showed protection by several antibiotics, including CAZ, against the action of stimulated PMN on lymphoblastoid cells (Daudi cell line), and attributed this effect to inactivation of HOCl produced extracellularly by MPO. Preliminary results from our laboratory suggest that CAZ also protects endothelial cells from the cytolytic activity of activated PMN.

We thus see that CAZ, in addition to its antibiotic activity possesses antioxidant properties.

These could be useful in situations where excessive PMN activation, production of activated species of oxygen, and liberation of MPO are seen. Sepsis and septic shock are two such situations. In our model, the lowest concentration associated with a protective effect ($10^{-5}$M = 5.46 μg/ml, 26% protection) is inferior to the serum concentrations attained in patients treated with this antibiotic: following an intravenous infusion of 2 g CAZ in healthy volunteers and patients, the serum concentration peaks at 59 to 83 μg/ml.[34] The combination of an antioxidant activity with those of an antibiotic in one molecule allows simultaneous treatment with a well characterized, widely used drug, of both the infectious aspect of the disease process, as well as the consequences of excessive PMN activation.

### References

1. Neuhof H. Actions and interactions of mediator systems and mediators in the pathogenesis of ARDS and multiorgan failure. *Acta Anaesthesiol Scand* 1991; **35(suppl 95):** 7–14.
2. Ciprolle MD, Pasquale MD, Cerra FB. Secondary organ dysfunction. From clinical perspective to molecular mediators. *Critical Care Clinics* 1993; **9(2):** 261–298.
3. Lamy M, Deby-Dupont G. Is sepsis a mediator-inhibitor mismatch? *Intensive Care Med* 1995; **21:** S8–S15.
4. Klebanoff SJ. Phagocytic cells: products of oxygen metabolism. In: Gallin JI, Goldstein IM, Snyderman R, eds. *Inflammation, basic principles and clinical correlates.* New York: Raven Press, 1988; 391–444.
5. Oddel EW, Segal AW. The bactericidal effects of the respiratory burst and the myeloperoxidase system isolated in neutrophil cytoplasts. *Biochim Biophys Acta* 1988; **971:** 266–274.
6. Klebanoff SJ, Hamon CB. Role of myeloperoxidase-mediated antimicrobial systems in intact leukocytes. *J Reticuloendothel Soc* 1972; **12:** 170–196.
7. Allen RC, Stjernholm RL, Steele RH. Evidence for the generation of an electronic excitation state(s) in human polymorphonuclear leukocytes and its participation in bactericidal activity. *Biochem Biophys Res Commun* 1972; **47:** 679–684.
8. Weiss SJ. Tissue destruction by neutrophils. *New Engl J Med* 1989; **320:** 365–376.
9. Matheson NR, Wong PS, Travis J. Enzymatic inactivation of human $\alpha_1$-proteinase inhibitor. *Biochem Biophys Res Comm* 1979; **88:** 402–409.
10. Reddy VY, Pizzo SV, Weiss SJ. Functional inactivation and structural disruption of human $\alpha_2$-macroglobulin by neutrophils and eosinophils. *J Biol Chem* 1989; **264:** 13801–13809.
11. Deby-Dupont G, Croisier J-L, Camus G, *et al.* Inactivation of $\alpha_2$-macroglobulin by activated human polymorphonuclear leukocytes. *Mediators of Inflammation* 1994; **3:** 117–123.
12. Ottonello L, Dallegri F, Dapino P, Pastorino G, Sacchetti C. Cytoprotection against neutrophil-delivered oxidant attack by antibiotics. *Biochem Pharmacol* 1991; **42:** 2317–2321.
13. Cantin A, Woods DE. Protection by antibiotics against myeloperoxidase-dependent cytotoxicity to lung epithelial cells *in vitro. J Clin Invest* 1993; **91:** 38–45.
14. Lapenna D, Cellini L, De Gioja S, *et al.* Cephalosporins are scavengers of hypochlorous acid. *Biochem Pharmacol* 1995; **49;** 1249–1254.
15. Deby-Dupont G, Mathy-Hartert M, Jadoul L, Vandenberghe A, Lamy M, Deby C. Ceftazidime: an antibiotic with a host defense spectrum interesting in cases of deleterious polymorphonuclear leukocytes (PMN) activation. In: *34th Interscience Conference on Antimicrobial Agents and Chemotherapy,* Orlando, Florida, USA, 4–7 October 1994; abst. G30, p42.
16. Mathy-Hartert M, Deby C, Deby-Dupont G, Vandenberghe A, Jadoul L, Lamy M. Mechanisms of antioxidant protection of endothelial cells against polymorphonuclear leucocyte oxidant stress by ceftazidime. *Clin Intens Care* 1994; **5(2 suppl):** 74.
17. Deby-Dupont G, Mathy-Hartert M, Deby C, Jadoul L, Vandenberghe A, Lamy M. Ceftazidime (CAZ) protects plasmatic antiproteases from oxidative inactivation. *Can J Infect Dis* 1995; **6(suppl C):** 425C, abstract 3207.
18. Jaffe EA, Nachman RL, Becker CG, Monick CR. Culture of human endothelial cells derived from human umbilical veins. *J Clin Invest* 1973; **52:** 2747–2753.
19. Bakkenist ARJ, Wever R, Vulsma T, Plat H, Van Gelder BF. Isolation procedure and some properties of myeloperoxidase from human leucocytes. *Biochem Biophys Acta* 1978; **524:** 45–54.
20. Mathy-Hartert M, Deby-Dupont G, Melin P, Lamy M, Deby C. Cultured macrophages acquire a bactericidal activity against *Pseudomonas aeruginosa* after incorporation of myeloperoxidase. *Experientia* (in Press).
21. *Worthington Enzyme Manual.* Freehold NJ, Worthington Biochemical Corp 1972; 43–45.
22. Hager LP, Morris DR, Brown FS, Eberwein H. Chloroperoxidase: utilisation of halogen anions. *J Biol Chem* 1966; **241:** 1769–1777.
23. Ager A, Gordon JL. Differential effects of hydrogen peroxide on indices of endothelial cell function. *J Exp Med* 1984; **159:** 592–603.
24. Bellus D. Physical quenchers of singlet molecular oxygen. *Adv Photochem* 1984; **11:** 105–205.
25. Ouannès C, Wilson T. Quenching of singlet oxygen by tertiary aliphatic amines. Effect of DABCO. *J Am Chem Soc* 1968; **90:** 6526–6528.
26. Zabucchi G, Soranzo MR, Menagazzi ER, Bertoncin P, Nardon E, Patriarca P. Uptake of human eosinophil peroxidase and myeloperoxidase by cells involved in the inflammatory process. *J Histochem Cytochem* 1989; **37:** 499–508.
27. Wasil M, Haliwell B, Moorhouse CP. Scavenging of hypochlorous acid by tetracycline, rifampicin and some other antibiotics: a possible antioxidant action of rifampicin and tetracycline? *Biochem Pharmacol* 1988; **37:** 775–778.
28. Fukase Y, Abe Y, Takahashi T, Ishikawa M. Effect of antibiotic administration on chemiluminescence and adherence of human neutrophils. *Chemotherapy* 1989; **37:** 1195–1199.
29. Gunther MR, Mao J, Cohen MS. Oxidant-scavenging activities of ampicillin and sulbactam and their effect on neutrophil functions. *Antimicrob Agents Chemother* 1993; **37:** 950–956.
30. Khan AU. Myeloperoxidase singlet molecular oxygen generation detected by direct infrared electronic emission. *Biochem Biophys Res Commun* 1984; **122:** 668–675.
31. Kanofsky JR. Biochemical requirements for singlet oxygen production by purified human myeloperoxidase. *J Clin Invest* 1984; **74:** 1489–1495.
32. Rawls HR, van Santen PJ. Singlet oxygen: a possible source of the original hydroperoxides in fatty acids. *Ann NY Acad Sci* 1970; **171:** 135–138.
33. Miyachi Y, Yoshioka A, Imamura S, Niwa Y. Effect of antibiotics on the generation of reactive oxygen species. *J Invest Dermatol* 1986; **86:** 449–453.
34. Rains CP, Bryson HM, Peters DH. Ceftazidime. An update of its antibacterial activity, pharmacokinetic properties and therapeutic effect. *Drugs* 1995; **49(4):** 577–617.

ACKNOWLEDGEMENTS. This work was supported by the FRSM (Fund for Medical Scientific Research—Belgium) grants no 3.4554.93 and 3.4556.95, and by the European Commission Concerted Action Contract BMH1-CT94-1249. The authors gratefully thank Dr G. Hartstein for his assistance with the English translation of this paper.

**Received 4 September 1995; accepted in revised form 16 October 1995**
